# The soybean gene Gm*Hsp*22.4 is involved in the resistance response to *Meloidogyne javanica* in *Arabidopsis thaliana*

**DOI:** 10.1186/s12870-020-02736-2

**Published:** 2020-11-24

**Authors:** Suellen Mika Hishinuma-Silva, Valéria Stefania Lopes-Caitar, Rafael Bruno Guayato Nomura, Bruna Caroline Sercero, Aline Garcia da Silva, Mayra Costa da Cruz Gallo De Carvalho, Ivani de Oliveira Negrão Lopes, Waldir Pereira Dias, Francismar Corrêa Marcelino-Guimarães

**Affiliations:** 1 Department of Biochemistry and Biotechnology, Londrina State University, Londrina, Brazil; 2 Department of Plant Sciences, University of Tennessee, Knoxville, TN USA; 3 Department of Production and Plant Protection, Agronomic Institute of Paraná-IAPAR, Londrina, Brazil; 4 Northern Paraná State University, Bandeirantes, Brazil; 5 Department of Plant Biotechnology, Brazilian Agricultural Research Corporation EMBRAPA Soybean, Londrina, PR Brazil

**Keywords:** Root-knot nematode, HSP, Defense response, Reproduction factor

## Abstract

**Background:**

Small heat shock proteins (sHSPs) belong to the class of molecular chaperones that respond to biotic and abiotic stresses in plants. A previous study has showed strong induction of the gene Gm*Hsp*22.4 in response to the nematode *Meloidogyne javanica* in a resistant soybean genotype, while repression in a susceptible one. This study aimed to investigate the functional involvement of this small chaperone in response to *M. javanica* in *Arabidopsis thaliana*. First, it was evaluated the activation of the promoter region after the nematode inoculation, and the occurrence of polymorphisms between resistant and susceptible re-sequenced soybean accessions. Then functional analysis using *A. thaliana* lines overexpressing the soybean Gm*Hsp*22.4 gene, and knocked-out mutants were challenged with *M. javanica* infestation.

**Results:**

High expression levels of the GFP gene marker in transformed *A. thaliana* plants revealed that the promoter region of Gm*Hsp*22.4 was strongly activated after nematode inoculation. Moreover, the multiplication of the nematode was significantly reduced in plants overexpressing Gm*Hsp*22.4 gene in *A. thaliana* compared to the wild type. Additionally, the multiplication of *M. javanica* in the *A. thaliana* mutants was significantly increased mainly in the event *athsp*22.0–2. This increase was not that evident in the event *athsp*22.0–1, the one that preserved a portion of the promoter region, including the HSEs in the region around − 83 bp. However, structural analysis at sequence level among soybean resistant and susceptible genotypes did not detect any polymorphisms in the whole gene model.

**Conclusions:**

The soybean chaperone Gm*Hsp*22.4 is involved in the defense response to root-knot nematode *M. javanica* in *A. thaliana*. Specifically, the promoter region covering until − 191 from the transcriptional start site (TSS) is necessary to promoter activation after nematode infection in Arabidopsis. No polymorphisms that could explain these differences in the defense response were detected in the Gm*Hsp*22.4 gene between resistant and susceptible soybean genotypes. Therefore, further investigation is needed to elucidate the triggering factor of the plant’s defense mechanism, both at the sequence level of the soybean genotypes presenting contrasting reaction to root-knot nematode and by detecting cis-elements that are essential for the activation of the Gm*Hsp*22.4 gene promoter.

**Supplementary Information:**

The online version contains supplementary material available at 10.1186/s12870-020-02736-2.

## Background

The increase in soybean productivity through genetic gains over the years is undeniable. However, the management of pests and pathogens still poses major challenges in soybean production either by its impact on the environment or by its burden in the production costs [1]. Among the pests, phytonematodes are found in all major soybean cultivation areas in Brazil, and worldwide. Production losses in soybean crops in Brazil caused by phytoparasitic nematodes were estimated to be around R$ 16.2 billion/year [2].

Several strategies have been developed to control phytonematodes, such as crop rotation, the use of nematicides and biological treatments [3, 4]. Additionally, transgenic approaches involving the overexpression and silencing of genes have been attempted [5, 6]. Finally, genetic resistance has been used in genetic breeding programs exploring genetic *loci* in soybean, such as the quantitative trait loci (QTLs) described in plant introductions (PIs) [7]. However, a few sources of resistance are available, limiting the development of broad durable resistance [8]. Thus, understanding the molecular mechanisms involved in the resistance response is an important approach for the development of biotechnological strategies for the control of these pests [9].

Chaperone proteins are present in both prokaryotes and eukaryotes and are widely distributed in several species in the plant kingdom. In plants, biotic and abiotic stresses can trigger diverse defense mechanisms, such as the activation of a group of highly conserved proteins known as heat shock proteins (HSPs) [10]. The main function of the HSPs is to act as molecular chaperones, performing maintenance of the spatial structure of other proteins that are negatively affected by changes in factors such as temperature [11, 12]. These proteins were initially observed in the salivary glands of *Drosophila* spp. under heat shock stress [11]. Based on their sequence size and homology, the HSPs have been grouped into five classes: HSP60, HSP70, HSP90, HSP100 and HSP20 (small-HSP or sHSP). HSP20 proteins exhibit an N-terminal hydrophobic region that is quite divergent in its sequence and length in different proteins, followed by a conserved domain of approximately 90–100 amino acid residues in the C-terminal part of the protein and a short C–terminal extension [13–16].

Despite their name, HSP20 proteins are induced not only after thermal shock, but also by other abiotic stimuli, such as water deficits, heavy metals, ozone and UV radiation [17], as well as under different biotic stresses, such as nematode infestation [15, 17, 18]. Studies on the functions of cytosolic HSP20 proteins have suggested that HSP20 maintains the remaining cellular proteins in an active state under stressful conditions via the linking of its dissociated dimers with denatured proteins [13, 16, 19, 20]. According to this hypothesis, the heat-induced dissociation of HSP20 could lead to exposure of the hydrophobic region and, consequently, to the stabilization of the denatured proteins [15, 16]. HSP20 then cooperates with other ATP-dependent molecular chaperones such as HSP70, HSP90, HSP100 and GroEL to refold proteins [14, 16]. In addition, HSP20 exhibits a much higher binding stoichiometry than other molecular chaperones, leading to some speculation that HSP20 functions as a reservoir to stabilize the flow of denatured proteins in response to stress [15, 16].

In a previous study, Lopes-Caitar et al. [21] characterized the expression profiles of 51 members of the HSP20 family in *Glycine max* (Gm*Hsp*20) under abiotic (heat and cold) and biotic stresses (infestation by *M. javanica*) on susceptible (BRS 133) and resistant (PI595099) soybean genotypes. The expression levels of these genes were strongly dependent on the genotype under biotic stress conditions. Additionally, the exposure period (four or 8 days after inoculation) played a significant role in the gene expression in both genotypes. Five out of the 51 members of the Gm*Hsp*20 family were significantly expressed under both treatments, but the expression of the Gm*Hsp*22.4 (abbrev. for Glyma10g176400) gene stood out. Its relative expression level was 60 times higher in the infected resistant genotype compared to the false inoculated samples, while repression was observed in the susceptible genotype in the presence of the nematode. Interestingly, the authors reported the systematic occurrence of a standard organization of cis-elements in the promoter region of these soybean family genes associated with the responses of soybean to *M. javanica* infestation.

In this study, we sought to elucidate the involvement of Gm*Hsp*22.4 in response to *M. javanica* infestation. We first evaluated the promoter activation under the presence of the root-knot nematode. Then, in silico and functional analyses were performed to characterize the function of the chaperone Gm*Hsp*22.4 on the response to *M. javanica*’s infestation. Our results provide important data about the role of this gene in the defense response against this nematode.

## Results

### The Gm*Hsp*22.4 promoter is strongly induced by *M. javanica* in Arabidopsis roots

**Fig. 1:**
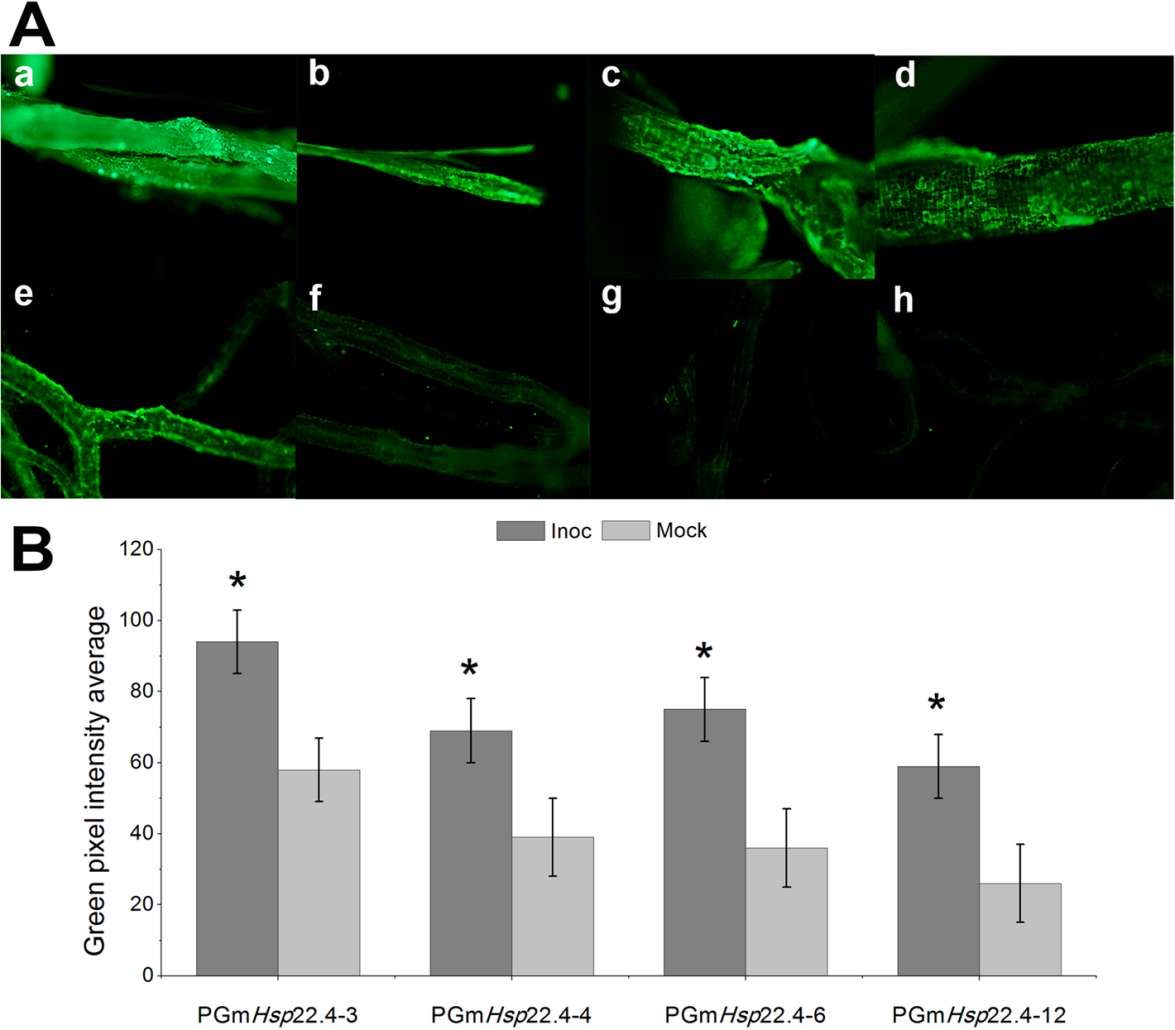
Expression of GFP protein in transgenic *Arabidopsis thaliana* roots. **A** *Arabidopsis thaliana* roots of 4 stable transgenic events were observed and photographed under a fluorescence microscope in not inoculated (mock) or inoculated (inoc) at 9 days with *Meloidogyne javanica*. Green fluorescence indicates expression from the promoter and induction of the GFP marker. **a** PGm*Hsp*22.4–3 inoc. **b** PGm*Hsp*22.4–4 inoc. **c** PGm*Hsp*22.4–6 inoc. **d** PGm*Hsp*22.4–12 inoc. **e** PGm*Hsp*22.4–3 mock. **f** PGm*Hsp*22.4–4 mock. **g** PGm*Hsp*22.4–6 mock. **h** PGm*Hsp*22.4–12 mock. **B** Quantification of the levels of green fluorescence of the four events of transgenic *Arabidopsis thaliana*, analyzed by the Adobe® Photoshop® CS6 13.0 × 32 software program. The data shown are representative of three biological replicates for each condition (inoc and mock). * indicates statistical significance at the 5% level (Scheffé’s -test) compared to the mock control

To investigate the responsiveness of the Gm*Hsp*22.4 promoter to *M. javanica* infestation, the potential Gm*Hsp*22.4 promoter sequence (2 Kb from the transcriptional start site - TSS) was fused with the coding region of GFP and introduced into *A. thaliana* seedling, resulting in four events (PGm*Hsp*22.4–3, 4, 6 and 12)*.* The fluorescence levels in the roots of these events were measured at the 9th day after inoculation (dai) with *M. javanica* and in non-inoculated plants (mock). As expected, the average GFP activity was significantly higher in all tested events (PGm*Hsp*22.4–3, 4, 6 and 12) than in the mock events (Fig. 1).

### Structural and polymorphism analysis of Gm*Hsp*22.4 in *M. javanica* resistant and susceptible genotypes

Gm*Hsp*22.4 gene presented a predicted total length of 1158 bp, being 337 bp in the 5 ‘UTR region, 588 bp in the coding region and 233 bp in the 3’ UTR region; with no introns, this sequence resulted in a potential encoded protein of 196 aa. The orthologous gene in *A. thaliana* exhibited a similar organization, presenting 74.6% similarity with the Gm*Hsp*22.4, also with no intron region and potentially encoding a protein of 196 aa.

Based on the resequencing data obtained by Santos et al. [22] of 21 soybean accessions, a potential promoter region spanning 2.0 kb from the TSS and the complete transcriptional region (including exons and UTRs) were compared between six soybean genotypes resistant to *M. javanica* and 15 susceptible ones [8]. The Gm*Hsp*22.4 gene was highly conserved in all 21 genotypes evaluated, with no sequence variation. Similarly, the promoter region showed no sequence variation, except for a substitution in the − 1229 position (A/T) upstream of the transcription start site, but it was not possible to associate this substitution with resistance to the pest, as it was randomly distributed among the genotypes.

### Overexpression of Gm*Hsp*22.4 increases resistance to root-knot nematodes in Arabidopsis

**Fig. 2:**
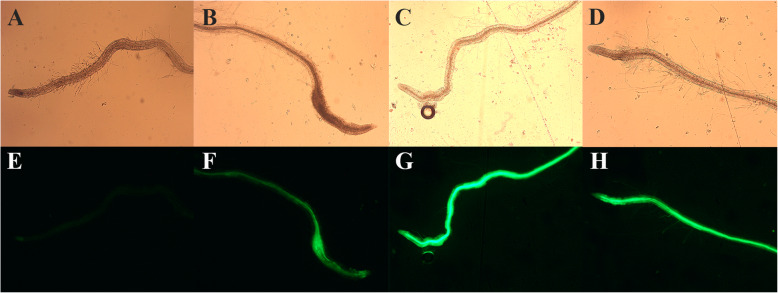
GFP expression in transgenic *Arabidopsis thaliana* roots overexpressing Gm*Hsp*22.4. The roots were observed and photographed under a Zeiss Axio Scope A1 optical microscope using a common light with no filter (**a-d**) and a GFP filter (E-H). **a** WT (OE) no filter. **b** Gm*Hsp*22.4–4-OE no filter. **c** Gm*Hsp*22.4–6-OE no filter. **d** Gm*Hsp*22.4–8-OE no filter. **e** WT (OE) GFP filter. **f** Gm*Hsp*22.4–4-OE GFP filter. **g** Gm*Hsp*22.4–6-OE GFP filter. **h** Gm*Hsp*22.4–8-OE GFP filter

*Agrobacterium tumefaciens* carrying the Gm*Hsp*22.4::pH7WG2D construct was used to transform *A. thaliana*. Three stable events overexpressing the coding region of Gm*Hsp*22.4 under 35S promoter control were obtained by floral dip transformation. Positive events were selected and confirmed by the detection of eGFP fluorescence in *A. thaliana* roots. Strong green fluorescence was observed in the roots of three homozygous events in the T4 generation, demonstrating the success of the transformations with Gm*Hsp*22.4, while in the wild-type (WT) no fluorescence was detected (Fig. 2).

Root morphology attributes (length and weight) of the transformed plants were compared with the wild-type counterparts, to detect possible changes caused by the overexpression of the Gm*Hsp*22.4. The average root length and weight of the transgenic lines was 8.0 cm and 60 mg respectively, which was not significantly different of the wild-type Scheffé’s test (*p* ≤ 0.05). These data and other morphological comparisons are described in the Additional file 1**.**

**Fig. 3:**
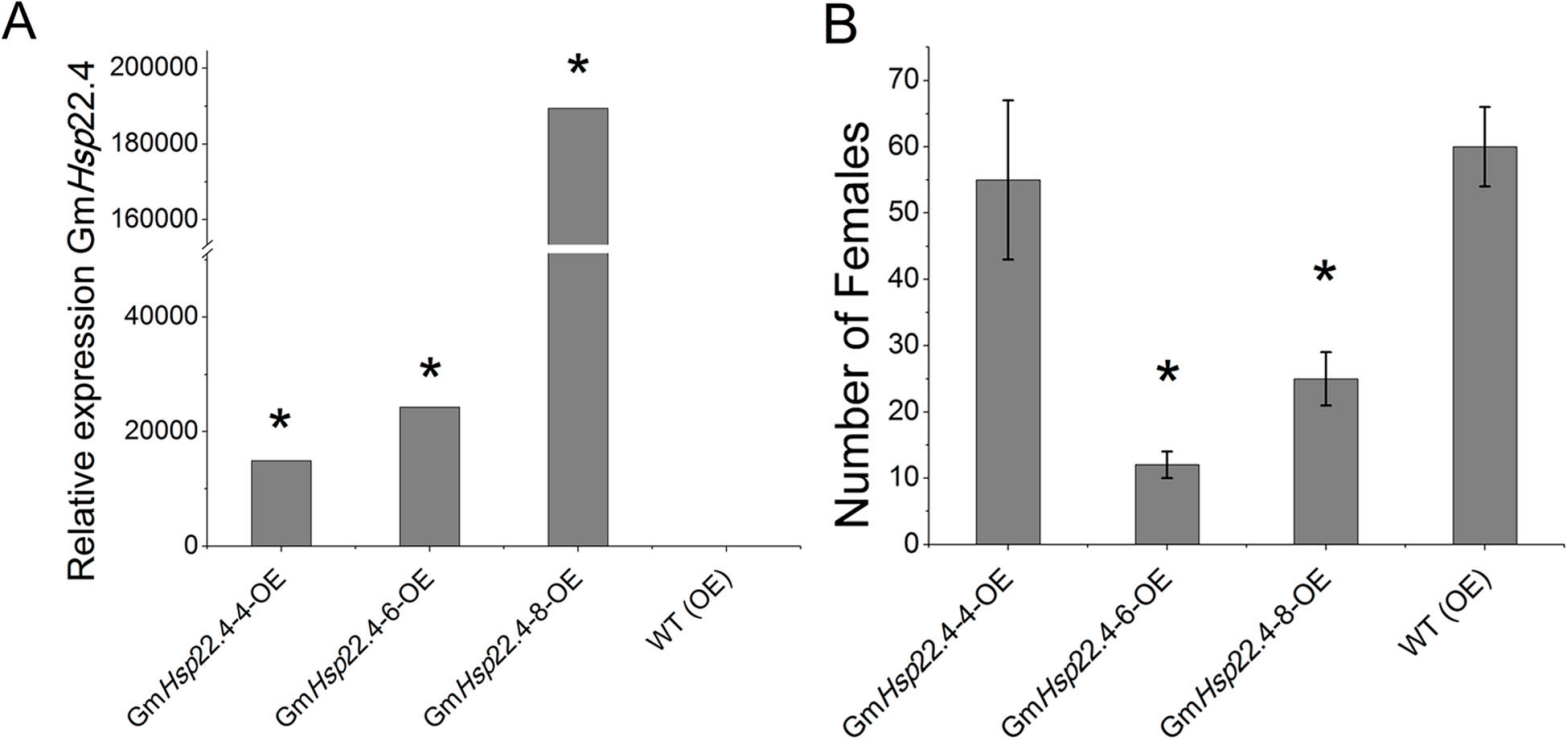
Evaluation of expression and numbers of *Meloidogyne javanica* females. **a** Relative expression of Gm*Hsp*22.4 in transformed and WT *Arabidopsis thaliana* plants infected with *Meloidogyne javanica*. **b** Number of *Meloidogyne javanica* females at 22 days after inoculation in Gm*Hsp*22.4-overexpressing and WT *Arabidopsis thaliana* (*n* = 14). Data are expressed as the mean ± standard error of the mean. A *p*-value ≤0,05. * indicates statistical significance at the 5% level (Scheffé’s -test) compared to WT

The expression levels of the Gm*Hsp*22.4 gene in the 14 transgenic plants for each event were quantified by RT-qPCR. As expected, high levels of gene expression were detected in *A. thaliana* plants transformed with the target gene regulated by the 35S promoter (Fig. 3a). The highest expression was observed in the Gm*Hsp*22.4–8-OE line, followed by the Gm*Hsp*22.4–6-OE and Gm*Hsp*22.4–4-OE events, respectively.

The effect of Gm*Hsp*22.4 overexpression in *A. thaliana* transgenic plants against *M. javanica* infestation was examined by counting the number of *M. javanica* females on plant roots compared to wild-type plants at 22 dai (Fig. 3b). Among the three homozygous events tested, a significant reduction in the number of females was observed for the Gm*Hsp*22.4–6-OE (82%) and Gm*Hsp*22.4–8-OE (42%) events when compared to the WT (OE). The Gm*Hsp*22.4–4-OE event did not result in a significant reduction in the number of *M. javanica* females (Fig. 3b).

### Knockout of the Gm*Hsp*22.4 orthologue in Arabidopsis compromises the defense response to *M. javanica*

To confirm the involvement of soybean Gm*Hsp*22.4 in the response to nematode infestation, two knock-out *A. thaliana* mutants for the orthologous gene were tested against pest infestation. The *athsp*22.0–1 and *athsp*22.0–2 mutants consisted of a T-DNA insertion in the promoter at − 191 pb upstream the TSS or in the 5′ UTR at + 149 pb downstream the TSS, respectively. The homozygous lines of the mutants were selected by PCR (Additional file 2 and Additional file 5).

The effects of the mutations on plant growth and development were verified by comparing the root length (cm), root mass (mg), number of leaves and mass of the fresh aerial part (mg) of the events *athsp*22.0–1 and *athsp*22.0–2 against the corresponding attributes in the WT (Additional file 3). No significant differences were detected between the averages of these morphological parameters from transgenic and WT plants. The average root lengths of the transgenic plants were equal to 11.4 cm, while 11.2 cm for WT. The root mass for the transgenic plants were 835.3 (*athsp*22.0–1) and 938.2 (*athsp*22.0–2) milligrams, while in the WT it was 976.4 mg. Likewise, no significant differences were found between the number of leaves and the shoot weight in transgenic events and wild type plants. Therefore, the evaluated morphological parameters did not indicate any significant difference in the development of the transgenic and wild-type *A. thaliana* plants.

**Fig. 4:**
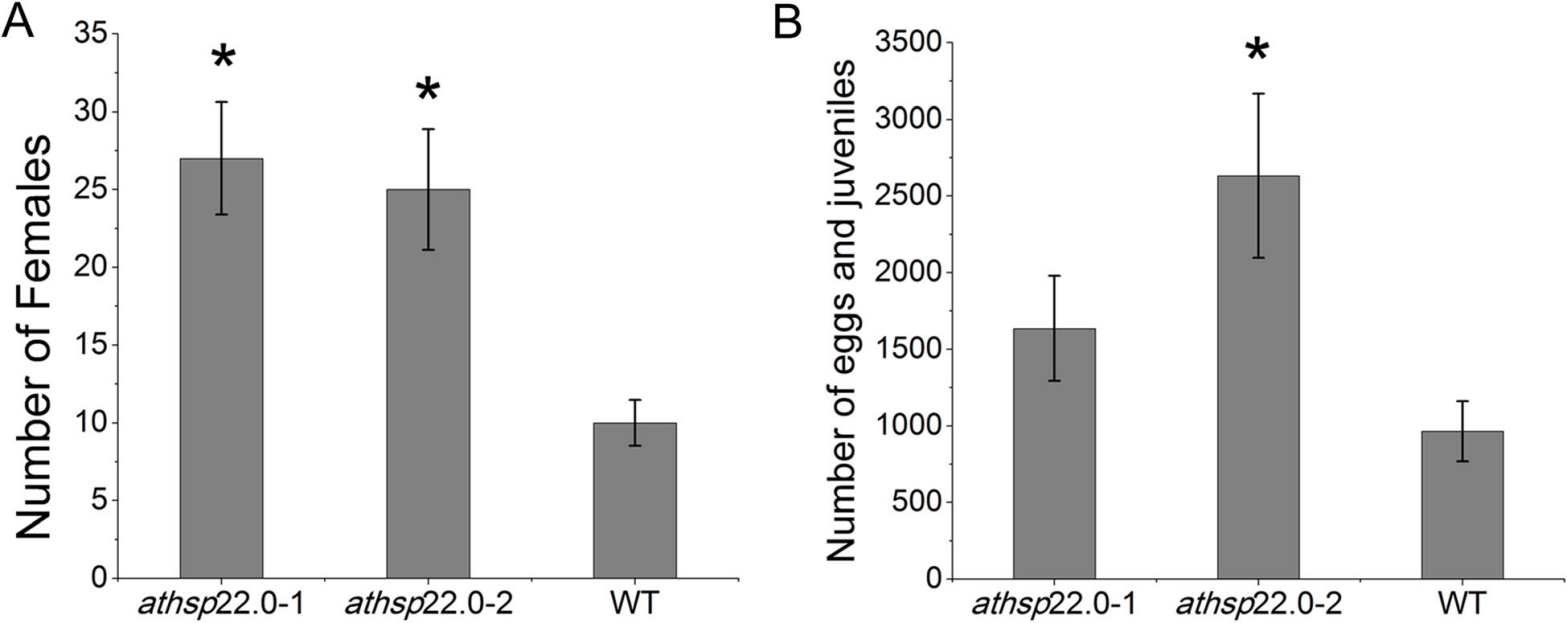
Resistance of the knockout mutant. *athsp*22.0–1 and *athsp*22.0–2 plants were compared to WT plants under infestation with *Meloidogyne javanica* (*n* = 20). **a** Number of female nematodes per plant at 45 day after inoculation (dai). **b** Number of eggs / juveniles per plant at 45 dai. Data are expressed as the mean ± standard error of the mean. * indicates statistical significance at the 5% level (Scheffé’s -test) compared to WT

Finally, the effect of the mutations on the nematode’s ability to infect and multiply on *A. thaliana* plants was evaluated through the number of eggs, juveniles (J2) and females obtained in plants of the two transgenic events and in the WT inoculated with *M. javanica*. The number of females associated with both events at 45 dai showed a significant increase in the multiplication of approximately 150% compared to the WT (Fig. 4a). On the other hand, the number of juveniles/eggs was significantly higher only in the *A. thaliana athsp*22.0–2 mutant (2631.15), when compared with the control (964.68), being the number of juveniles/eggs in the mutant *athsp*22.0–1 (1636.29) (Fig. 4b).

Based on these results, the *athsp*22.0–1 and *athsp*22.0–2 *A. thaliana* mutants showed increased susceptibility to *M. javanica,* being the latter the one who presented significantly higher numbers of both females and eggs and juveniles than the wild-type plants Scheffé’s test (*p* ≤ 0.05).

### Analysis of cis-elements in the promoter region of the Arabidopsis At4g10250 gene

**Fig. 5:**
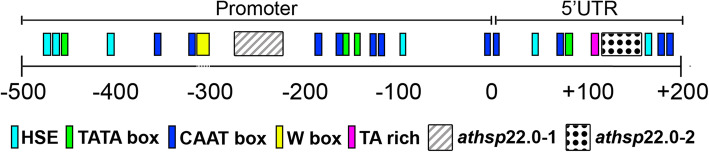
Schematic map of the locations of different cis elements in the At4g10250 gene in both promoter and 5′ UTR regions. PlantCARE and AthaMap were used to analyze the region 500 bp upstream of the transcription start site. The dark blue boxes represent CAAT boxes; the light blue boxes represent HSEs; the green boxes represent TATA boxes; the pink box represents the TA-rich element; and the yellow box represents the W-box. *dotted line: reverse strand. The *athsp22.0–1* represent the T-DNA insertion site in the promoter region and *athsp22.0–2* in 5′ UTR region

Based on the analysis of the promoter region of At4g10250, we identified a TA-rich sequence at the position of + 114 bp and TATA boxes at + 79, − 146, − 165 and − 456 bp of the TSS. The CAAT box elements were found in the sequence at positions + 182, + 173, + 71, + 5, − 2, − 121, − 126, − 165, − 181, − 310 and − 355 bp of the TSS. The Heat Shock Elements (HSEs), which are recognized and activated by the heat shock factors (HSF) transcription factors [23], were observed at six different positions, + 166, + 48, − 97, − 408, − 462 and − 467 bp of the TSS. In contrast to the other elements, a W-box was located in the negative strand at the − 303 bp position (Fig. 5).

The T-DNA insertion lines were localized between the CAAT-box and W-box cis-elements in the promoter region of the event *athsp*22.0–1, and between the cis-element HSE and TA-rich in the 5′ UTR of the event *athsp*22.0–2 (Fig. 5).

## Discussion

As chaperones, sHSP proteins have been described as induced by different biotic and abiotic stress, including nematode infection [12, 14, 15]. In this study, we examined specifically the role of the Gm*Hsp*22.4 gene in the *M. javanica* resistance response in details. This gene was selected because a previous study reported it to be strongly expressed in the soybean *M. javanica*-resistant genotype PI 595099 and repressed in the susceptible genotype BRS 133, when they were exposed to biotic and abiotic stresses [21]. The soybean accession has been described as an important source of resistance against specific strains and races of nematode species, including *M. javanica,* and contains a QTL (SOYHSP 176) mapped on soybean chromosome 13 involved in the resistance [24–27].

Our results demonstrated that Gm*Hsp*22.4 promoter activity in *A. thaliana* plants is highly activated after nematode infestation, confirming the transcriptional activation in response to *M. javanica* previously reported [21]. The Gm*Hsp*22.4 promoter presents a structural organization in which CAAT boxes are located immediately upstream of HSE elements, a W-box located further upstream of the HSEs [21]. This conserved promoter structure was found in *GmHsp20* family members responsive to nematode infestation, where the HSEs are potentially recognized by heat shock transcription factors [21]. As expected, promoter induction was on average 34.47% greater in the infected Arabidopsis transgenic plants than in the non-infected ones, as revealed by GFP fluorescence marker (Fig. 1).

Although we demonstrated the activity of the promoter of the Gm*Hsp*22.4 in *M. javanica* responses, we could not demonstrate how such different levels of Gm*Hsp*22.4 transcripts and the associated contrasting phenotypes of resistant and susceptible soybean accessions are explained at the gene sequence level. Our analysis of 21 re-sequenced soybean accessions did not detect any variation in the promoter, 5’UTR, 3’UTR or exons of the Gm*Hsp*22.4 gene that correlated with the phenotype. The whole gene and promoter regions were identical in the resistant and susceptible materials evaluated.

The differential regulation of the expression of Gm*Hsp*22.4 between the resistant and susceptible genotypes was in accordance with the results obtained by Fuganti et al. [27]. These authors mapped a QTL related to root-knot nematode resistance in a population derived from the resistant source PI595099 between two microsatellite markers, Satt 144 and Soy*HSP* 176. The Soy*HSP* 176 marker was located in a region containing another sHSP, the Gm*Hsp*17.6-L gene. Additionally, the expression levels of this gene were found to be differentially regulated between resistant and susceptible individuals from the mapped population, having been induced only in resistant individuals. A polymorphism analysis of the promoter region of the gene Gm*Hsp*17.6-L by Fuganti et al. [18] detected a greater number of AT (n) repeats in the resistant genotype, compared to the susceptible one, whereas those numbers were constant in the gene Gm*Hsp*22.4 in genotypes analyzed in this work. Therefore, our analysis did not indicate any correlation between AT(n) repetitions and promoter activity for the gene Gm*Hsp*22.4.

One possible explanation to the differential expression of the gene Gm*Hsp*22.4 in phenotypically contrasting reactions of soybean genotypes to *M. javanica* infection in our study could be the existence of enhancer regions that interacting with promoter regions of other genes, either in their vicinity or over long distances, altering their regulation [28]. However, our data was not sufficient to confirm this hypothesis.

Another explanation is epigenetic regulation, which cannot be detected at a sequence level. This mechanism has been reported to be involved in soybean resistance to cyst nematodes [29]. In another study [30], differential levels of methylation were observed in soybean plants after soybean cyst nematode (SCN) infestation, which may affect the accessibility of transcription factors to cis-elements regulating the transcriptional activity of genes responsive to the nematode [31]. Although this scenario may be an alternative explanation for the regulation of Gm*Hsp*22.4, further studies are necessary to confirm this hypothesis.

Arabidopsis presents 19 genes encoding *Hsp20*s*,* grouped into 12 subfamilies based on their subcellular localization and homology [21], being the transcript of the *A. thaliana* gene model orthologous to Gm*Hsp*22.4, At*Hsp*22.0, undetectable in normal conditions (22 °C) and cumulative to high levels in response to heat stress (38 °C) [32]. To date, there is no available evidence of the activation of At*Hsp*22.0 under biotic stress conditions, such as nematode infestation.

To obtain a better understanding of the roles of this chaperone in the resistance response to root-knot nematodes, we overexpressed the soybean gene in *A. thaliana* plants and studied two DNA insertion lines in which the orthologous genes were knocked-out. In our study, two events presenting constitutive overexpression of the Gm*Hsp*22.4 gene in *A. thaliana* resulted in a significant reduction in the numbers of females of 82 and 42% (Fig. 3b). Similarly, when the orthologous gene was knocked out in Arabidopsis plants, we observed an increase of 150% in the number of females (Fig. 4a). Based on these results, we conjecture that Gm*Hsp*22.4 is involved in nematode infestation responses, possibly acting as the first line of cellular defense by capturing unfolded proteins and reducing protein aggregates sizes. Thus, with the generation of more binding sites, and the assistance of ATP-dependent HSP70 and HSP100, aggregation is reversed and refolding is facilitated [33]. The high transcript levels of Gm*Hsp*22.4 upon its overexpression in transformed plants may have improved the stability of plant proteins in the endoplasmic reticulum, either by favoring the defense response or by maintaining the supply of active chaperones in the plant during pest infestation.

Considering the susceptibility levels of the mutant lines related with the position of the T-DNA insertion in the promoter region, we observed a less severe impact in the event *athsp*22.0–1, where the number of eggs and juveniles were not different from the wild plants (Fig. 4b). In this mutant, a larger portion of the promoter was not affected by the T-DNA insertion (− 191 from TSS), where the organizational structure of nematode responsive promoter was maintained, including the HSE element around − 83 pb from the TSS (Fig. 5). On the other hand, the T-DNA insertion in *athsp*22.0–2 in the 5′ UTR (+ 149 position) completely eliminated the resistance, confirming the importance of this HSE element region close to the TSS in the Gm*Hsp*22.4 orthologous after nematode infestation.

The role of *Hsp*20 related to its nematode-responsive promoter activity has been described in rice by Escobar et al. [34], who characterized the involvement of the soybean Hs*Hsp*17.4 gene in the response to infestation by *M. incognita*. The authors also observed that the promoter of the Hs*Hsp*17.4, a rice sHSP, was able to induce the expression of β-glucuronidase (GUS) marker gene and, consequently, 50 to 70% galls in the roots were stained after 17 to 20 days after infestation with *M. incognita*. In addition, they observed that mutations in the 83 bp region upstream from the TSS are determinant for the promoter activation in the nematode response [34], corroborating our results.

Similarly, Barcala et al. [17] described the importance of combinations and/or specific sequences of HSEs for regulation in different situations. Functional analysis of the promoter regions of Ha*Hsp*17.6G1 and Ha*Hsp*18.6G2, other two sHSP in sunflower, also associated the promoter organization with the ability to respond to nematode infection. Only Ha*Hsp*18.6G2 was induced in giant cells, which presents two HSEs in the promoter, one of which was proximal and the other was distant. In contrast, only one HSE was observed in the Ha*Hsp*17.6G1 promoter, in a distant region. It was also found that the CAAT box element in *HaHsp18*.6G2 was located immediately upstream and between the HSEs, and only downstream of the HSE in the *HaHsp17*.6G1 promoter.

Interestingly, it was observed an organization of the soybean gene promoters responsive to nematode infestation similar to that of the cis-elements of the At4g10250 promoter as previously described by Lopes-Caitar et al. [21]. The promoters of the soybean Hsp20 genes responsible for *M. javanica* infestation presented two CAAT elements in the region containing the HSE element in their structures, while the W-box was located farther away [21]. On the other hand, it was not possible to observe the occurrence of an HSE element within the − 83 bp region from the TSS in At4g10250, but it was closely located at − 97 bp position from the TSS. Thus, similar structural organization of *Hps22.*4 between soybean and Arabidopsis could also reflect functional conservation.

## Conclusions

Although the Gm*Hsp*22.4 gene was initially identified as being highly induced in a *M. javanica* nematode-resistant soybean genotype and repressed in a susceptible one, no differences in polymorphism were observed, either in the promoter or in the coding regions of those genotypes. The Gm*Hsp*22.4 gene affects the resistance response to *M. javanica*, since its overexpression reduced the infective potential of nematodes by up to 82% and Arabidopsis ortholog knock-out lines increased the susceptibility by 150%. The promoter of the Gm*Hsp*22.4 gene was induced in response to infestation with *M. javanica* at 9 dai, but no difference between the susceptible and resistant plant promoters were detected. A promoter region of At*Hsp*22.0 containing at least 191 bp, with the presence of HSE close to the TSS, is necessary to trigger the resistance of *A. thaliana* to *M. javanica* nematode.

## Methods

### Nematode culture and plant materials

The population of *M. javanica* was multiplied in a greenhouse of the Nematology Department at Embrapa Soja, Londrina, Parana State, Brazil, in soybean plants and processed according to the technique of J Bonetti and J Ferraz [35]. In a hatching chamber, the viable second stage J2 were collected every day, for 3 days, in an Erlenmeyer flask and was quantified using a Peter’s chamber.

Seeds of soybean accession PI 595099 used in this study were provided by Soybean Germoplasm Bank from Embrapa Soybean, Londrina, Parana State, Brazil.

Seeds of *Arabidopsis thaliana* ecotype Columbia (Col-0) and seeds of *Arabidopsis thaliana* of two independent lines from a single knockout mutant of *AtHsp*22.0 were obtained from the Arabidopsis Biological Resource Center (ABRC) (WiscDsLox489_492E13 with stock number N858258 for *athsp*22.0–1 and GK-265F12–014990 with stock number N335081 for *athsp*22.0–2). No specialist undertook the formal identification the plant materials used in our study.

### Polymorphism analysis of the Gm*Hsp*22.4 gene

Resequencing data of 21 soybean genotypes were obtained from Santos et al. [22]. The fasta file for the nucleotide sequence spanning the interval of 41,003,835 to 41,006,424 was uploaded, based on the soybean genome (https://phytozome.jgi.doe.gov/pz/portal.html**#**, version Wm82.a1.v1).

The phenotypic responses of the materials to *M. javanica* infestation were obtained at the Embrapa Soja-Nematology Laboratory [8], with BRS Valiosa RR, BRSGO 8360, CD 201, MG/BR46 Conquista, PI 595099 and Paraná, being classified as resistant cultivars, and Anta 82, BR 16, BRS 232, BRS 360RR, BRS 361, BRS Sambaíba, BRSGO Chapadões, BRSMT Pintado, BRSMT Uirapuru, FT Abyara, FT Cristalina, IAS 5, NA 5909 RG, P98Y11 and Williams82 as susceptible.

The nucleotide sequences of all 21 genotypes were evaluated with Integrative Genomics Viewer (IGV) [36].

### Cloning of the Gm*Hsp*22.4 gene from soybean

The DNA of the *M. javanica* nematode-resistant soybean genotype PI595099 was extracted from leaf tissue according to the technique described by Doyle and Doyle [37] and used for the amplification of the coding region with the primers CDSGm*Hsp*22.4-F and CDSGm*H*sp22.4-R, while the promoter region was amplified with the primers PGm*Hsp*22.4-F and PGm*Hsp*22.4-R (Additional file 5). The PCR fragments were purified using the Wizard®SV gel and PCR Clean-UP System (Promega).

**Fig. 6:**
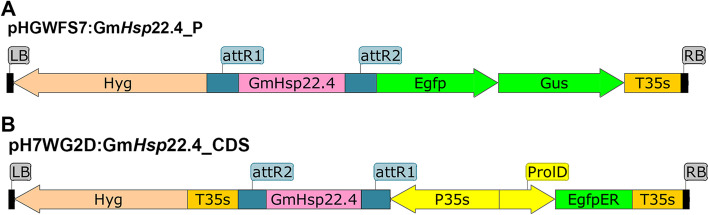
Scheme of the T-DNA regions of binary vectors. The vectors were used for Gm*Hsp*22.4_CDS overexpression and analysis of Gm*Hsp*22.4_P promoter activity in Arabidopsis. **a** Construct used for promoter analysis, pHGWFS7::Gm*Hsp*22.4_P. **b** Overexpression construct, pH7WG2D::Gm*Hsp*22.4_CDS. RB (RightBorder) and LB (LeftBorder), used for the transformation of plants. p35S, cauliflower mosaic virus (CaMV) 35S promoter, a highly active promoter in plants. attR1 and attR2, recombination sites. T35S, cauliflower mosaic virus terminator. Egfp encodes green fluorescence protein (GFP). Gus encodes the β-glucuronidase protein. Hyg indicates resistance to the antibiotic hygromycin

The fragments were introduced into an input vector with the PCR™8/GW/TOPO® TA Cloning® Kit (2012), which was then used for the transformation of cells of the electrocompetent strain *Escherichia coli* DH5α by electroporation. The selection of the transformed clones was performed through the use of the antibiotic hygromycin at a final concentration of 100 μg.mL-1 and enzyme analysis, while the recombinants and the clones containing the fragments in the expected orientation were determined by restriction reactions. The plasmid DNA from the clones containing the promoter and coding regions of Gm*Hsp*22.4 in the expected orientation were used in a recombination reaction with the vector pHGWFS7::P [38] for promoter analyses (Fig. 6a) and the vector pH7WG2D::CDS for coding region analyses (Fig. 6b) [38].

### *A. tumefaciens* GV3101 and plant transformation

The plasmids pHGWFS7 and pH7WG2D containing the correctly cloned fragments of interest were used to transform *A. tumefaciens* GV3101 strains by electroporation at 2,2 kV, 25 μF, with 1 wrist controller at 200 or 400 Ω. Plates containing YEP medium with gentamicin and hygromycin were incubated overnight at 28 °C. For the confirmation of positive bacterial clones, PCR was performed using the primer set PGm*Hsp*22.4-F and PGm*Hsp*22.4-R for the promoter and the primer set pH7WG2D-F and pH7WG2D-R for the coding region (Additional file 5). The recombinant bacteria were used to transform the *A. thaliana* Columbia (Col-0) ecotype, using the floral dip method [39]. The selection of transformed seeds in T0 and the subsequent T2, T3 and T4 generations was performed in medium containing 1 / 2x MS medium (Sigma Chemicals n°m-5519), 0.8% agar (Sigma Chemicals n° A-1296), and 15 μg / ml-1 of hygromycin. Transformants were identified as hygromycin-resistant seedlings when they did not present growth retardation. Positive events were confirmed via PCR (Additional file 4) and then propagated until the T4 lineage to be used in subsequent experiments.

### Molecular analysis of putative transgenic plants

The *A. thaliana* plants transformed with Gm*Hsp*22.4 and selected with the antibiotic hygromycin at 15 μg mL^− 1^ were confirmed by PCR using genomic DNA extracted from the roots (Additional file 4). The primer pairs PGm*Hsp*22.4-F and PGm*Hsp*22.4-R (promoter region) or pH7WG2D-F and pH7WG2D-R (coding region) were used for PCR according to the instructions of the *Taq* DNA Polymerase Invitrogen Kit. DNA extracted from untransformed plants were used as negative samples. The PCR conditions for the promoter region were 95 °C for 4 min, followed by 35 cycles of 95 °C for 30 s, 56 °C for 30 s and 72 °C for 45 s and one additional step of 72 °C for 5 min.

In the case of *A. thaliana* transformed with the Gm*Hsp*22.4 coding region, the PCR conditions were 95 °C for 4 min, followed by 35 cycles of 95 °C for 30 s, 62 °C for 30 s and 72 °C for 45 s, and an additional step at 72 °C for 5 min. Both amplification products were visualized by conventional electrophoresis in the translucent and photodocumentador (Loccus) equipment was used to acquire and store the images.

### Detection of GFP activity under *M. javanica* infestation

The *A. thaliana* transgenic lines PGm*H*sp22.4–3, PGm*H*sp22.4–4, PGm*H*sp22.4–6, and PGm*H*sp22.4–12, harboring PGm*H*sp22.4:GFP were used to determine the promoter activity of Gm*Hsp*22.4 on the basis of green fluorescence intensity. The evaluation was performed in the roots of plants infected with 400 J2 *M. javanica* individuals after 9 days of inoculation or left uninfected at 26 °C [21]. The experiment was conducted in a completely randomized design (CRD) with three plants and four independent transformation events. Images of roots expressing eGFP were captured with a 5.0 megapixel camera connected to a Zeiss Axio Scope A1 compound microscope (Zeiss Corporation) and processed with MOTIC software (version 2.0). Quantitative analysis of fluorescence levels was performed by using the Adobe® Photoshop® CS6 13.0 × 32 program. The statistical analyzes were performed using SAS/STAT® software, Version 9.4. Copyright© 2016 SAS Institute Inc. The contrasts between the means of treatments and controls were tested using the Scheffé’s test, at 5% significance level, in variance analysis models.

### Bioassays with *M. javanica*

For the functional analysis, 14 non transformed plants and plants transformed with the Gm*Hsp*22.4 coding region, overexpressing the GFP marker, were selected for a nematode bioassay conducted in a CRD. The population of *M. javanica* was multiplied, and the roots were challenged with 400 *M. javanica* J2 per plant. The inoculum were pipetted through a small open hole next to the root of the plant.

After 22 days of inoculation at 26 °C [40], the roots were collected and weighed individually. Evaluation of the number of females of *M. javanica* was performed by counting the nematodes stained with acid fuchsine according to [41]. All parameters evaluated were compared between wild-type plants and those transformed with the Gm*Hsp*22.4 gene. The statistical analyzes were performed using SAS/STAT® software, Version 9.4. Copyright© 2016 SAS Institute Inc. The contrasts between the means of treatments and controls were tested using the Scheffé’s test, at 5% significance level, in variance analysis models.

### Quantification of Gm*Hsp*22.4 transcript levels

Total RNA was extracted using the TRIzol reagent (1 mL / 100 mg tissue) as recommended by the manufacturer (Invitrogen). After extraction, the RNA samples were treated with deoxyribonuclease I (Kit Invitrogen - DNase I) according to the manufacturer’s recommendations to eliminate any DNA molecules present in the sample. The treated RNA was employed in the cDNA synthesis step using the SuperScript III Kit (Invitrogen) as recommended by the manufacturer. RT-qPCR was conducted in a StepOnePlus™ System thermocycler (Thermo Fisher Scientific) using the SYBR® Green PCR Master Mix kit (Applied Biosystems) according to the manufacturer’s instructions. For normalization, the AT_Act (actin At3g18780) gene was employed, which was amplified with the primers AT_Act-F and AT_Act-R, while the primer set RT-Gm*Hsp*22.4-F and RT-Gm*Hsp*22.4-R were used to the target gene (Additional file 5). Relative quantification of the target gene calculated by the REST program used wild-type control plants as calibrators for each corresponding treatment. The samples were evaluated with at least 14 replicates from each transgenic line, from which three technical replicates were used in the PCR.

### Assay of T-DNA insertion mutants

**Fig. 7:**
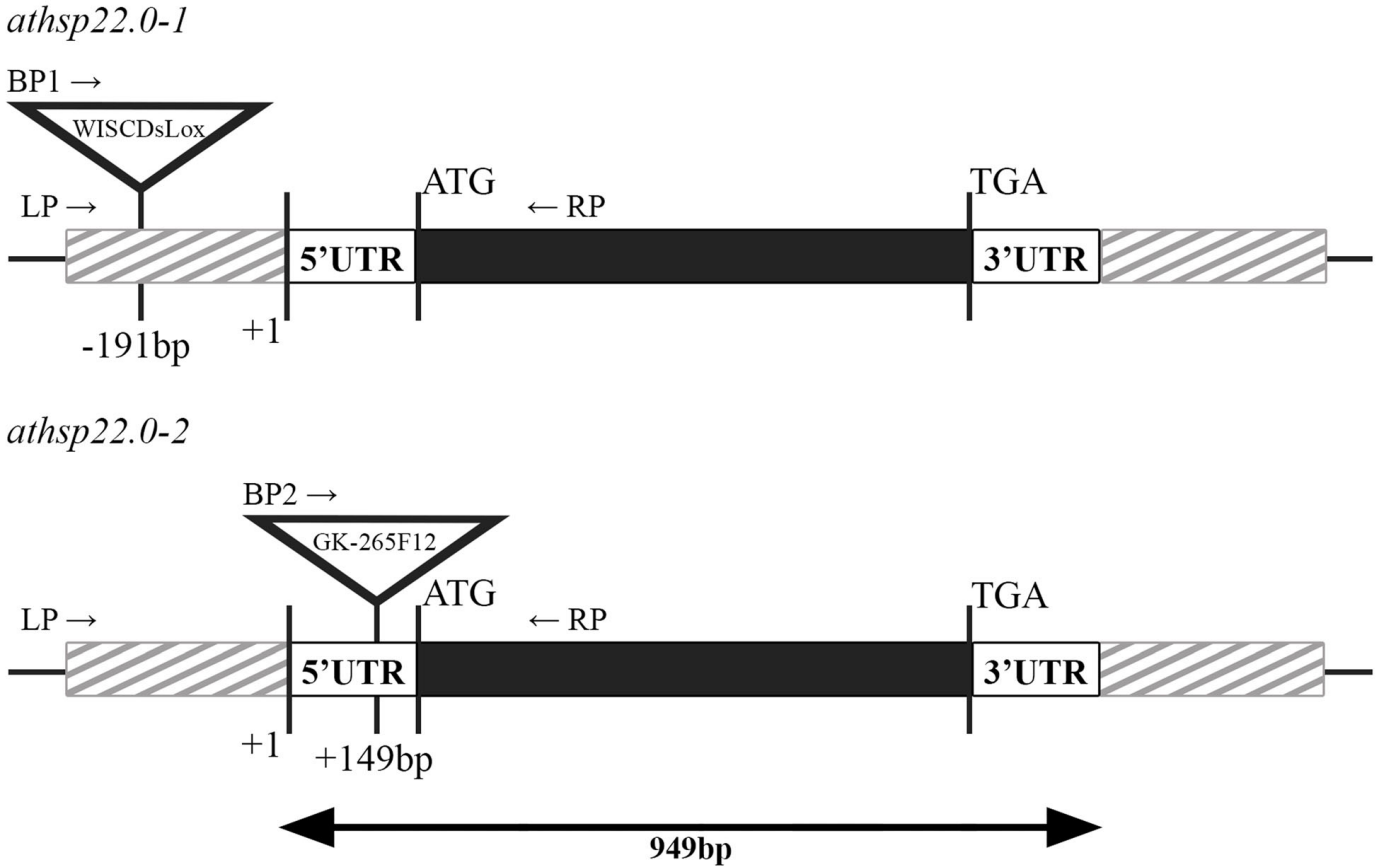
A scheme of the *Arabidopsis thaliana* At*Hsp*22.0 gene. Dark boxes represent the coding region, and striped gray boxes represent the promoter and terminator sequences. T-DNA insertion sites are indicated for both mutations (WiscDsLox for *athsp*22.0–1 and GK_265F12 for *athsp*22.0–2), and LP, RP, BP1 and BP2 indicate the localizations of the primers used for genotyping (Additional file 5)

Two independent lines from a single knockout mutant of *AtHsp*22.0 were obtained from the Arabidopsis Biological Resource Center (ABRC) (WiscDsLox489_492E13 with stock number N858258 for *athsp*22.0–1 and GK-265F12–014990 with stock number N335081 for *athsp*22.0–2). The codes *athsp*22.0–1 and *athsp*22.0–2 were adopted across the manuscript. Sequence analysis revealed that the T-DNA of *athsp*22.0–1 was inserted at 191 bp upstream relative to the transcript initiation site, while in the *athsp*22.0–2 mutant it was at 149 bp downstream (Fig. 7). Regarding the size of the T-DNA insert, the *athsp*22.0–1 was 8954 bp in length, and that of *athsp*22.0–2 was 6498 bp in length.

The homozygosity of these two mutants was verified using the primer pairs LP/RP and BP1/RP for *athsp*22.0–1 and LP/RP and BP2/RP for *athsp*22.0–2 (Additional file 5). One of the amplification reactions involved the use of the LP (left border) and RP (right border) primers, which surround the region flanking the insert, so it was only possible to verify amplification in the control plants, while amplification was not observed in the mutant plants due to the size of the insert. On the other hand, in the reaction using the primer RP (right border) as well as the primers BP1 and BP2, which anneal to the T-DNA in the promoter and exons, respectively, resulted in amplification only in mutant plants, as the control plants did not present the insertion.

After 45 days, the total number of females and eggs/juveniles stained with fuchsine acid [41] was evaluated according to Coolen and D’ Herde [42]. The two evaluated parameters were compared between the control plants and the mutants under a CRD. The statistical analyzes were performed using SAS/STAT® software, Version 9.4. Copyright© 2016 SAS Institute Inc. The contrasts between the means of mutants and controls were tested using the Scheffé’s test, at 5% significance level.

### Cis-element identification in the At4g10250 promoter

Putative cis-elements from the 500 bp region upstream of the transcript start site of the At4g10250 gene were characterized using the bioinformatic tool PlantCARE [43] and AthaMap [44] databases.

## Supplementary Information

**Additional file 1. **Phenotype of *Arabidopsis thaliana* overexpressing Gm*Hsp*22.4 and WT. **(A)** Root length, **(B)** Root weight, **(C)** Number of leaves. **(D)** Leaf area, **(E)** Number of flowers, **(F)** Weight of seeds, **(G)** Number of silique and **(H)** Shoot length. Data are expressed as the mean ± standard error of the mean. * indicates statistical significance at the 5% level (Scheffé’s -test) compared to WT.

**Additional file 2. **Molecular characterization of the *Arabidopsis thaliana* knockout mutants, *athsp22.0–1* and *athsp22.0–2*. **(A)** PCR amplification of the *athsp22.0–1* mutant, with the LP, left primer. RP, right primer. BP1, primer of T-DNA *athsp22.0–1* left border. **(B)** PCR amplification of the *athsp22.0–2* mutant, with the LP, left primer. RP, right primer. BP2, primer of T-DNA *athsp22.0–2* left border.

**Additional file 3. **Morphological characterization of *athsp*22.0–1, *athsp*22.0–2 and WT (*n* = 20). **(A)** Root length in cm. **(B)** Root weight in mg. **(C)** Number of leaves. **(D)** Fresh aerial part weight in mg. Data are expressed as the mean ± standard error of the mean. No significant differences were found between event and WT (Scheffé’s -test, *p* ≤ 5%).

**Additional file 4. **Molecular confirmation of the Gm*Hsp*22.4 promoter and coding region inserts in *Arabidopsis thaliana*. **(A)** PCR amplification of the promoter Gm*Hsp*22.4 region of different events and WT. **(B)** PCR amplification of the Gm*Hsp*22.4 coding region of different events and WT. In both transformations, the images shown are representative of about 14 biological replicates for each condition. In the insertion with promoter region the size of the amplicon was 1076 pb, the insertion of the coding region was 1331 pb. M indicates the 1 kb plus DNA Ladder.

**Additional file 5.** Primers used in this study.

## Data Availability

The datasets used and/or analysed during the current study are available from the corresponding author on reasonable request.

## Abbreviations

ABRC: Arabidopsis Biological Resource Center
bp: Base pair
CaMV: Cauliflower mosaic virus
CRD: Completely randomized design
dai: Day after inoculation
GFP: Green fluorescent protein
GUS: β-Glucuronidase
HSE: Heat shock element
HSF: Transcription factors
Inoc: Inoculated
J2: Second-stage juveniles
Mock: Not inoculated
QTL: Quantitative trait loci
SCN: Soybean cyst nematode
sHSP: Small heat shock protein
TSS: Transcription start site
WT: Wild-type
