# Supplementary figures and images for: The soybean gene GmHsp22.4 is involved in the resistance response to Meloidogyne javanica in Arabidopsis thaliana
Source: BMC Plant Biol. 2020 Nov 24;20:535. doi: 10.1186/s12870-020-02736-2 (PMC7687995; doi:10.1186/s12870-020-02736-2)

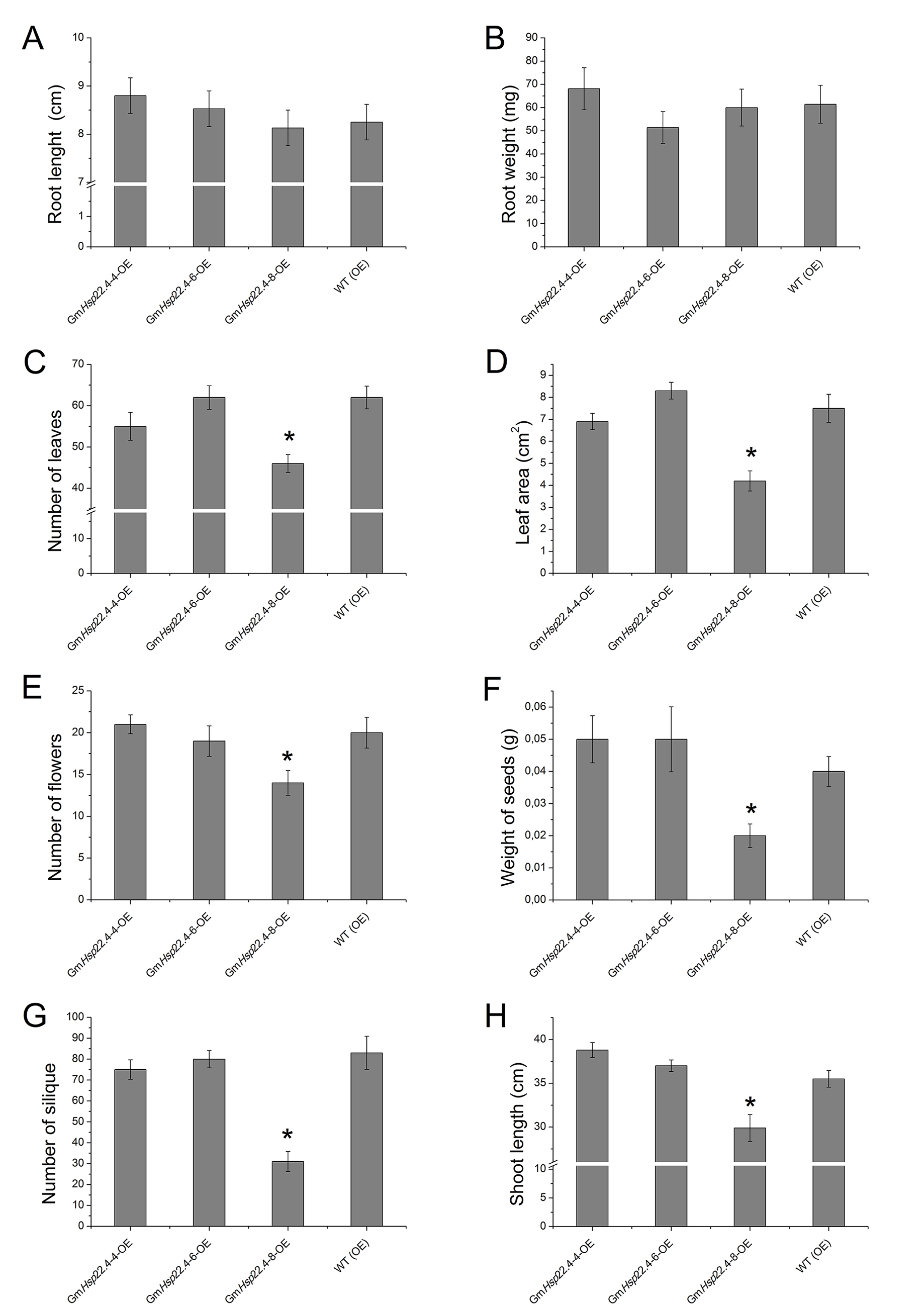

Supplement: Supplementary file 1 — Additional file 1. Phenotype of Arabidopsis thaliana overexpressing GmHsp22.4 and WT. (A) Root length, (B) Root weight, (C) Number of leaves. (D) Leaf area, (E) Number of flowers, (F) Weight of seeds, (G) Number of silique and (H) Shoot length. Data are expressed as the mean ± standard error of the mean. * indicates statistical significance at the 5% level (Scheffé’s -test) compared to WT. [file 12870_2020_2736_MOESM1_ESM.jpg]

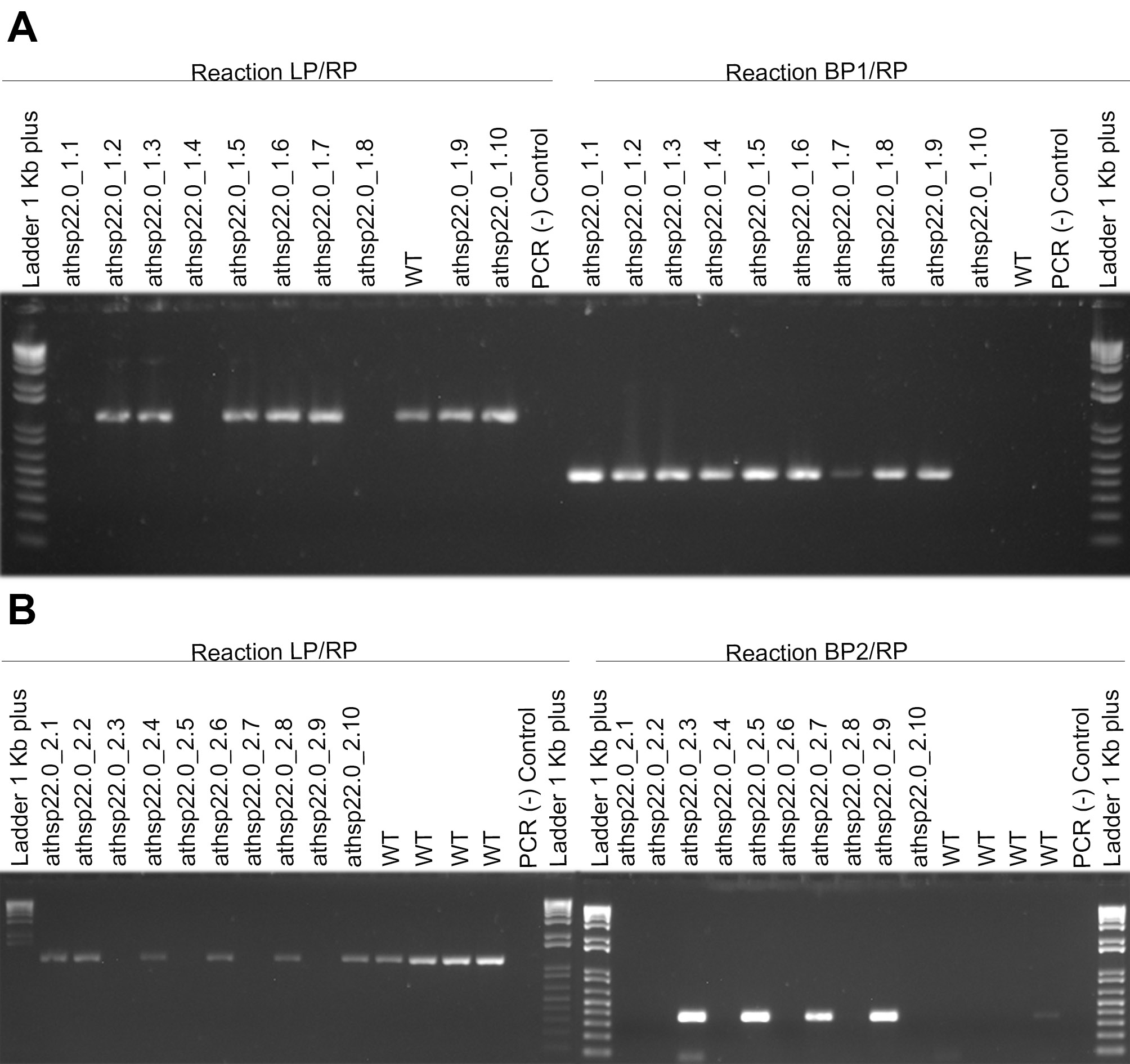

Supplement: Supplementary file 2 — Additional file 2. Molecular characterization of the Arabidopsis thaliana knockout mutants, athsp22.0–1 and athsp22.0–2. (A) PCR amplification of the athsp22.0–1 mutant, with the LP, left primer. RP, right primer. BP1, primer of T-DNA athsp22.0–1 left border. (B) PCR amplification of the athsp22.0–2 mutant, with the LP, left primer. RP, right primer. BP2, primer of T-DNA athsp22.0–2 left border. [file 12870_2020_2736_MOESM2_ESM.jpg]

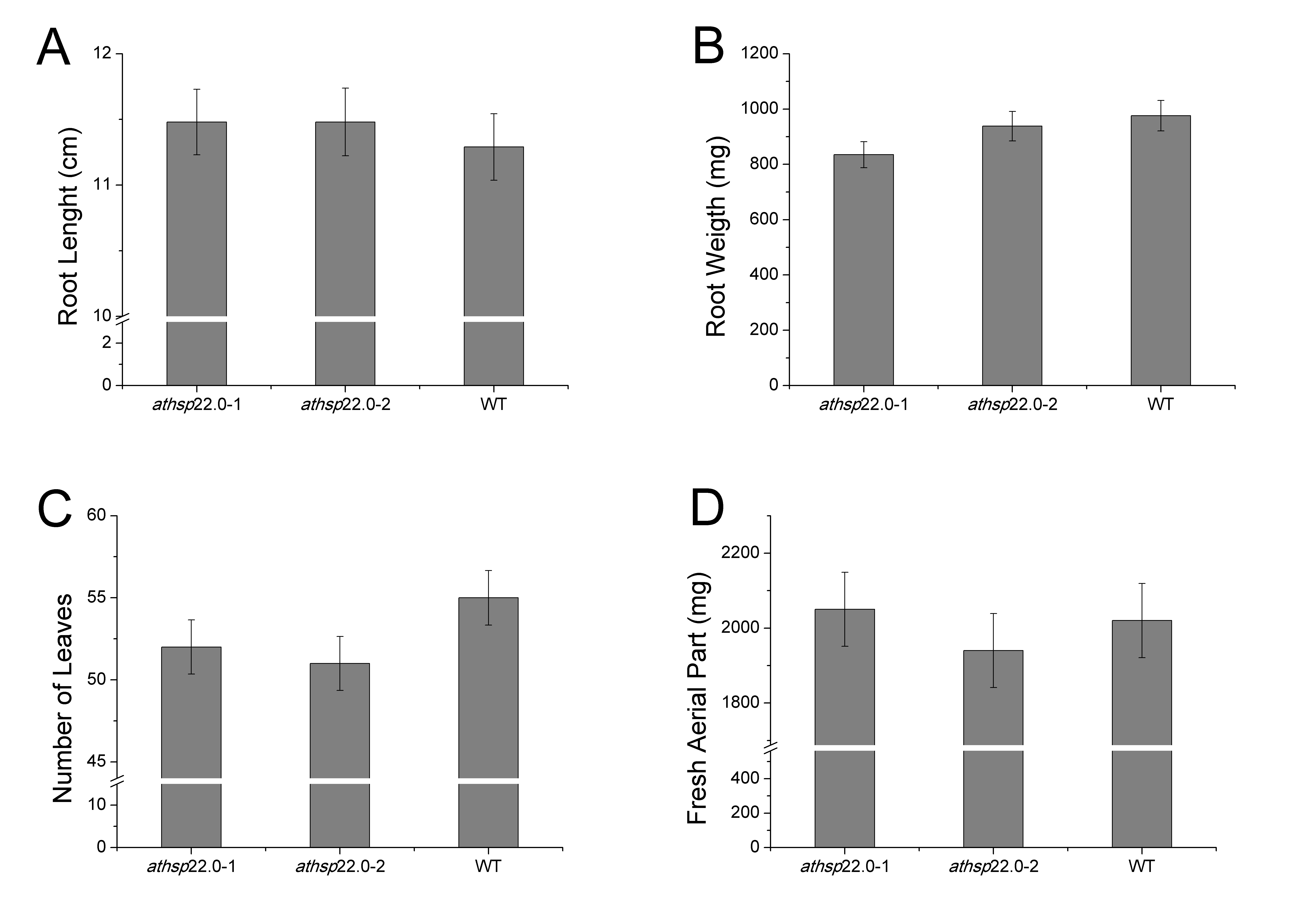

Supplement: Supplementary file 3 — Additional file 3. Morphological characterization of athsp22.0–1, athsp22.0–2 and WT (n = 20). (A) Root length in cm. (B) Root weight in mg. (C) Number of leaves. (D) Fresh aerial part weight in mg. Data are expressed as the mean ± standard error of the mean. No significant differences were found between event and WT (Scheffé’s -test, p ≤ 5%). [file 12870_2020_2736_MOESM3_ESM.jpg]

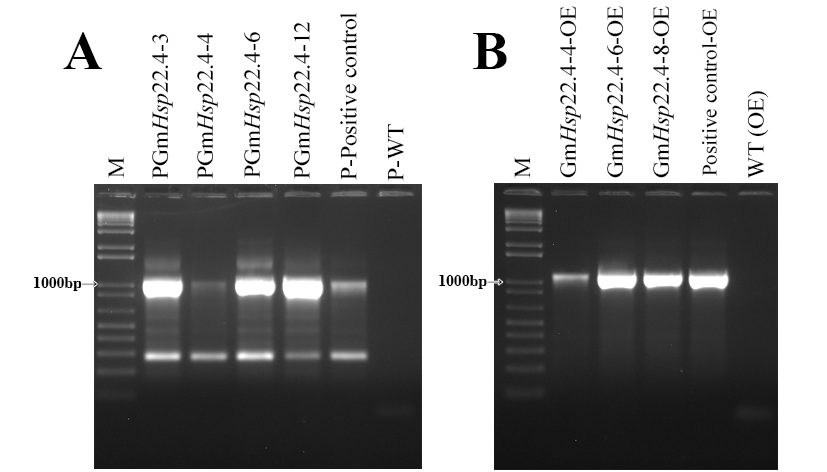

Supplement: Supplementary file 4 — Additional file 4. Molecular confirmation of the GmHsp22.4 promoter and coding region inserts in Arabidopsis thaliana. (A) PCR amplification of the promoter GmHsp22.4 region of different events and WT. (B) PCR amplification of the GmHsp22.4 coding region of different events and WT. In both transformations, the images shown are representative of about 14 biological replicates for each condition. In the insertion with promoter region the size of the amplicon was 1076 pb, the insertion of the coding region was 1331 pb. M indicates the 1 kb plus DNA Ladder. [file 12870_2020_2736_MOESM4_ESM.jpg]
